# Segmentation and Visual Analysis of Whole-Body Mouse Skeleton microSPECT

**DOI:** 10.1371/journal.pone.0048976

**Published:** 2012-11-12

**Authors:** Artem Khmelinskii, Harald C. Groen, Martin Baiker, Marion de Jong, Boudewijn P. F. Lelieveldt

**Affiliations:** 1 Division of Image Processing, Department of Radiology, Leiden University Medical Center, Leiden, The Netherlands; 2 Department of Nuclear Medicine, Erasmus MC, Rotterdam, The Netherlands; 3 Department of Radiology, Erasmus MC, Rotterdam, The Netherlands; 4 Department of Intelligent Systems, Delft University of Technology, Delft, The Netherlands; University of Navarra, Spain

## Abstract

Whole-body SPECT small animal imaging is used to study cancer, and plays an important role in the development of new drugs. Comparing and exploring whole-body datasets can be a difficult and time-consuming task due to the inherent heterogeneity of the data (high volume/throughput, multi-modality, postural and positioning variability). The goal of this study was to provide a method to align and compare side-by-side multiple whole-body skeleton SPECT datasets in a common reference, thus eliminating acquisition variability that exists between the subjects in cross-sectional and multi-modal studies. Six whole-body SPECT/CT datasets of *BALB/c* mice injected with bone targeting tracers ^99m^Tc-methylene diphosphonate (^99m^Tc-MDP) and ^99m^Tc-hydroxymethane diphosphonate (^99m^Tc-HDP) were used to evaluate the proposed method. An articulated version of the MOBY whole-body mouse atlas was used as a common reference. Its individual bones were registered one-by-one to the skeleton extracted from the acquired SPECT data following an anatomical hierarchical tree. Sequential registration was used while constraining the local degrees of freedom (DoFs) of each bone in accordance to the type of joint and its range of motion. The Articulated Planar Reformation (APR) algorithm was applied to the segmented data for side-by-side change visualization and comparison of data. To quantitatively evaluate the proposed algorithm, bone segmentations of extracted skeletons from the correspondent CT datasets were used. Euclidean point to surface distances between each dataset and the MOBY atlas were calculated. The obtained results indicate that after registration, the mean Euclidean distance decreased from 11.5±12.1 to 2.6±2.1 voxels. The proposed approach yielded satisfactory segmentation results with minimal user intervention. It proved to be robust for “incomplete” data (large chunks of skeleton missing) and for an intuitive exploration and comparison of multi-modal SPECT/CT cross-sectional mouse data.

## Introduction

Whole-body small animal imaging is widely used for the *in vivo* visualization of functional and anatomical information to study cancer, and for evaluation of drugs in pre-clinical research. An efficient combination of functional and structural information enables the visualization of cellular function and the follow-up of molecular processes in the living animals in their anatomical context. Functional information is provided by modalities such as Positron Emission Tomography (PET), Single Photon Emission Computed Tomography (SPECT), Magnetic Resonance Imaging (MRI) and Optical Imaging (OI), while anatomical information is usually obtained using Computed Tomography (CT) and ultrasound.

The data heterogeneity and volume created by whole-body multimodality imaging presents a complex problem with respect to combining, analyzing and quantifying this data with low inter-observer and intra-observer variability and minimal human input. This is caused in part by a high degree of shape and postural variability present in follow-up and cross-sectional animal studies. This variability is due to the fact that an animal body is a highly deformable system with many rigid parts (bones) and non-rigid structures (organs) [1], [2]. Also, there are no standardized protocols for animal positioning: if a subject is imaged using different imaging modalities and protocols during follow-up studies or if different animals are used, the subject is positioned in different ways and postural variations occur (*e.g.,* of the head, back and front limbs, *etc.*). One way to cope with this variability is to use multimodal animal holders between different scanners or use combined SPECT/CT, PET/CT, PET/MRI, or PET/SPECT/CT scanners that are becoming increasingly available. However, multimodal holders are not widely used or compatible and when they are, there are still significant differences in animal posture between different time points [3].

**Figure 1:**
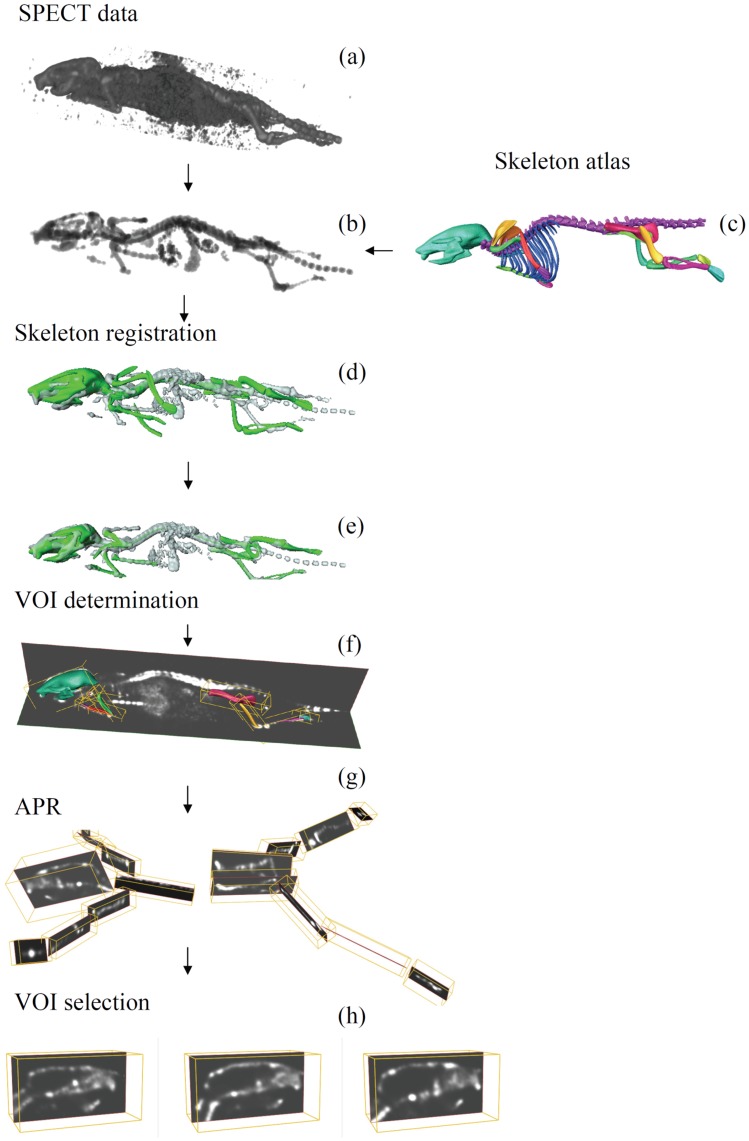
Overview of the proposed segmentation method. Given a SPECT dataset (a), the skeleton is extracted from the SPECT dataset (b). Next, the atlas skeleton (c) and the extracted skeleton (b) are registered to each other (d, e) using an anatomically realistic kinematic model. After the registration, the segmented data is reformatted into segments corresponding to the mouse atlas and thus mapping the data to a standardized atlas space (f, g). The data is now ready for an easy, fast and intuitive side-by-side exploration (multi-modal, follow-up or cross-sectional data) (h).

Various approaches were proposed to handle heterogeneous multi-modality data: Joshi *et al.* [4] proposed a method for fitting an elastically deformable mouse atlas to surface topographic range data acquired by an optical system; this method does not incorporate the extremities. Savinaud *et al.* [5] proposed a novel model-based approach to track animals in 3D from monocular video which allows the quantification of bioluminescence (BLI) signal on freely moving animals. Wildeman *et al.* [6] proposed a 2D/3D registration of µCT data to multiview photographs based on a 3D distance map combining optical/BLI data with CT. Suh *et al.* [7] published a serial registration method to serial µCT/SPECT mouse lower extremities images.

In [2], [3], the authors suggested the use of articulated whole-body small animal atlases as a standard geometric reference to tackle the problem of segmenting and organizing heterogeneous whole-body multi-modality small animal data. Using the articulated whole-body MOBY atlas, Baiker *et al.* presented a fully-automated skeleton registration and organ approximation method in low-contrast µCT mouse data [1]. This method exploits the high contrast of bone to automate the registration process of the skeleton model and the subsequent organ approximation. However, performing an anatomical CT scan together with a functional one is not always desired in longer term follow-up studies, where prolonged radiation exposure may become a confounding factor in cancer research, or may cause adverse radiation effects [8].

The goal of this study is to provide a segmentation and exploration tool for whole-body skeleton SPECT mouse data that eliminates any postural variability between the study subjects with minimal user intervention. Whole-body skeletal SPECT imaging with bone targeting tracers is of great interest for arthritis studies [9], development of bone pain palliation agents [10] as well in the field of bone metastases imaging in animal models. Since the location of metastatic appearance is unknown, whole-body scans, including follow-up, are essential to assess the growth and/or metastatic response to treatment [11].

This is a challenging task due to the nature of whole-body skeleton SPECT data: usually noisy, due to the relative short acquisition time and low resolution with an incomplete skeleton image (several portions missing in limbs, skull, *etc.*).

The main technical contributions of this paper are twofold:

- We present a semi-automated atlas-based skeleton segmentation method for whole-body SPECT mouse data that requires minimal user input;
- Using the Articulated Planar Reformation (APR) algorithm [12], [13], we provide the user with an intuitive side-by-side comparison and exploration platform for multi-modal (SPECT/CT), cross-sectional and follow-up data in a standardized layout, independent on the position of the animal during acquisition.

**Figure 2:**
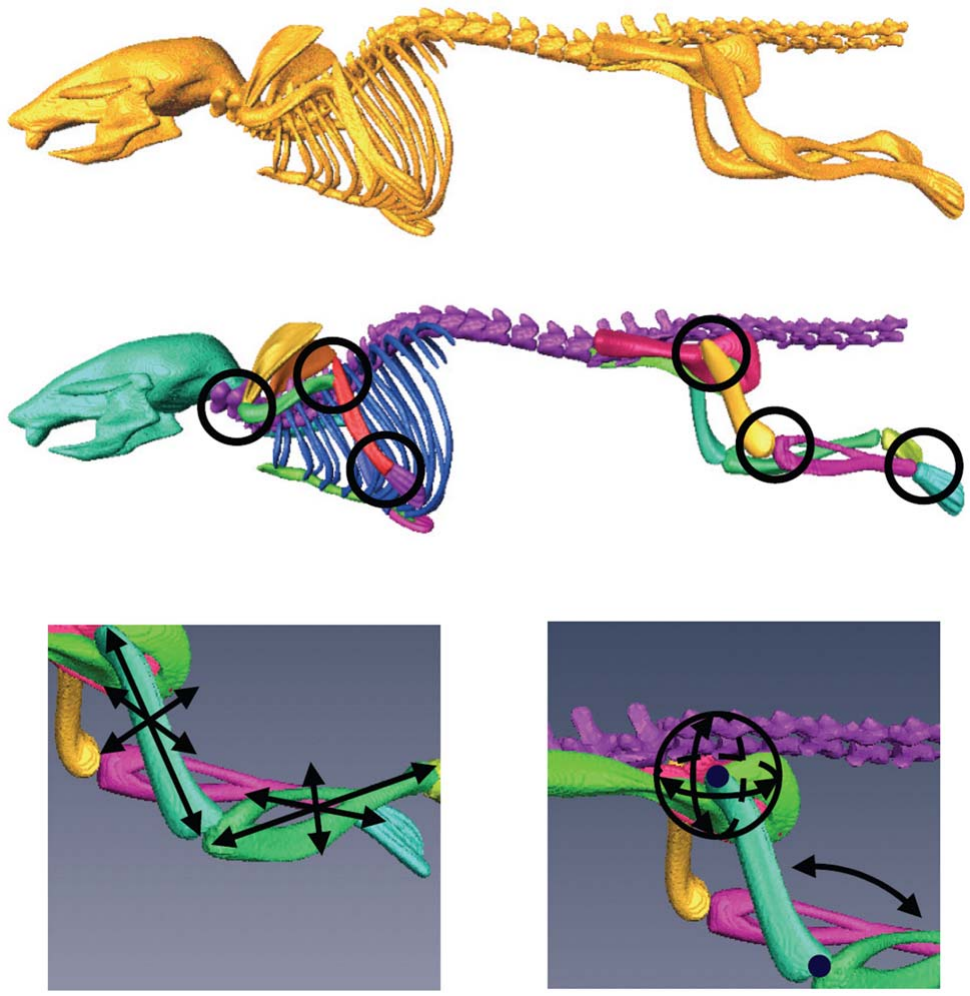
The MOBY mouse atlas skeleton. As originally included in the atlas (top), after segmenting the individual bones (middle), and a detail of the kinematic constraints and the DoFs of the femur/tibia-fibula bone complex (bottom). The colors indicate the different labels of each bone.

## Materials and Methods

### Method Overview

The first step of the proposed approach is to extract the skeleton from the SPECT data. Subsequently, the articulated mouse atlas is registered to the data following a hierarchical anatomical tree: first, the atlas is coarsely registered to the entire skeleton. Then, starting with the skull, each atlas bone is accurately registered to the correspondent bone in the data using the Iterative Closest Point (ICP) approach [14]. After the atlas is registered to the data, we apply the APR algorithm [12], [13] to reformat the segmented data into segments corresponding to a mouse atlas and thus mapping the data to a standardized atlas space. The presented method is validated using 6 *BALB/c* mice, and the quantitative performance of the method is assessed calculating the Euclidian point to surface distance between the atlas and the correspondent µCT skeleton surface. The results are compared to the results present in the literature for low-contrast µCT whole-body mouse data [1]. For a visual overview of the proposed method see Figure 1.

**Table 1:** Resolution of each SPECT and correspondent CT dataset.

	SPECT	CT
	Resolution (voxel size in mm^3^)	
Mouse 1	0.60×0.60×0.60	0.80×0.80×0.80
Mouse 2	0.30×0.30×0.30	0.99×0.99×1.00
Mouse 3	0.30×0.30×0.30	0.20×0.20×0.20
Mouse 4	0.30×0.30×0.30	0.10×0.10×0.10
Mouse 5	0.20×0.20×0.20	0.20×0.20×0.20
Mouse 6	0.20×0.20××0.20	0.10×0.10×0.10

**Figure 3:**
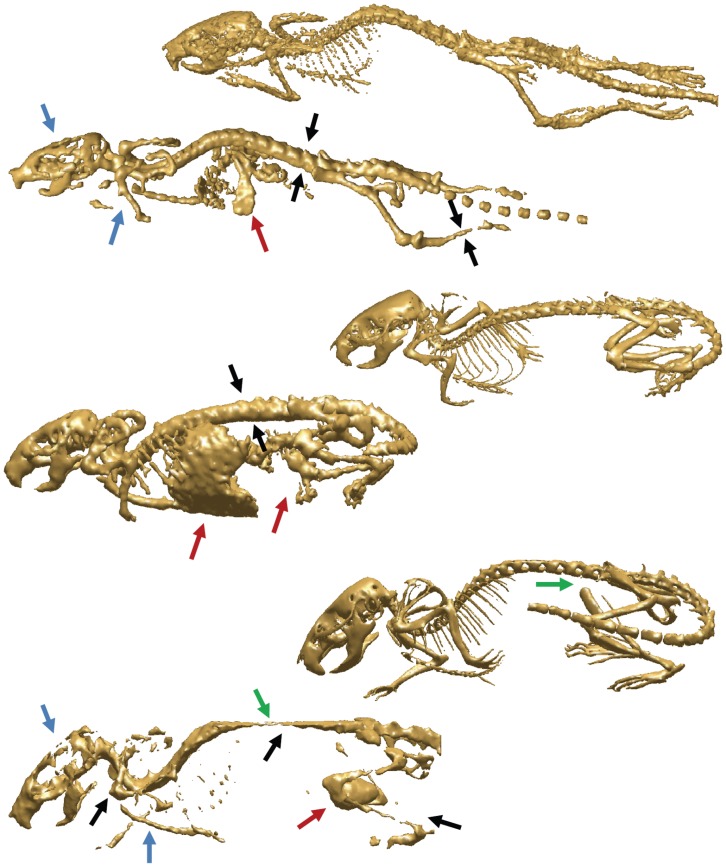
Examples of SPECT skeleton isosurfaces with the corresponding CT skeleton isosurfaces after the pre-processing step. The figure shows the positioning differences of the mouse in the scanner, SPECT (left) and the correspondent CT (right). The SPECT skeletons are incomplete, with several parts missing: especially in the case of front, hind limbs and the skull with large holes (blue arrows); also remnants of non-relevant objects such as lungs, kidneys and bladder are present (red arrows). In the bottom dataset the right femur and part of the spine are missing (green arrows) due to incomplete acquisition. The CT skeletons are complete and clean after the pre-processing step and are used in the validation of the proposed approach to calculate the Euclidean point to surface distance between the registered atlas and the skeleton surface. Black arrows indicate examples of regions where over and underestimation of the bone thickness occurred during the skeleton estimation in the data pre-processing step.

### Articulated MOBY Atlas

A realistic 4D digital mouse phantom was generated by Segars et al. [15] based on high-resolution 3D MRI data of a *C57BL/6*, 15 week old mouse from Duke University. The skeleton in this atlas did not distinguish between single bones and joints. To allow the registration to perform independent of the data acquisition protocol and large postural variations due to postural heterogeneity between scans, we presented a segmentation of the skeleton into individual bones and added anatomically realistic kinematic constraints and DoFs to each joint in [1], [3], [16]. Using the Amira™ V3.1 software [17] and guided by anatomical text books [18], [19] the following bones/bone groups were labeled: scapulae, humeri (upper front limbs), ulnae/radii (lower front limbs), manus (front paws), femora (upper hind limbs), tibiae/fibulae (lower hind limbs), pedes (hind paws), caput (skull), columna vertebralis (spine), costae (ribs), sternum (chest bone), and pelves. Each joint position was identified and the corresponding DoFs and kinematic constraints were specified. Two types of joints were distinguished: ball joints and hinge joints. The resulting articulated version of the MOBY skeleton can be seen in Figure 2.

**Figure 4:**
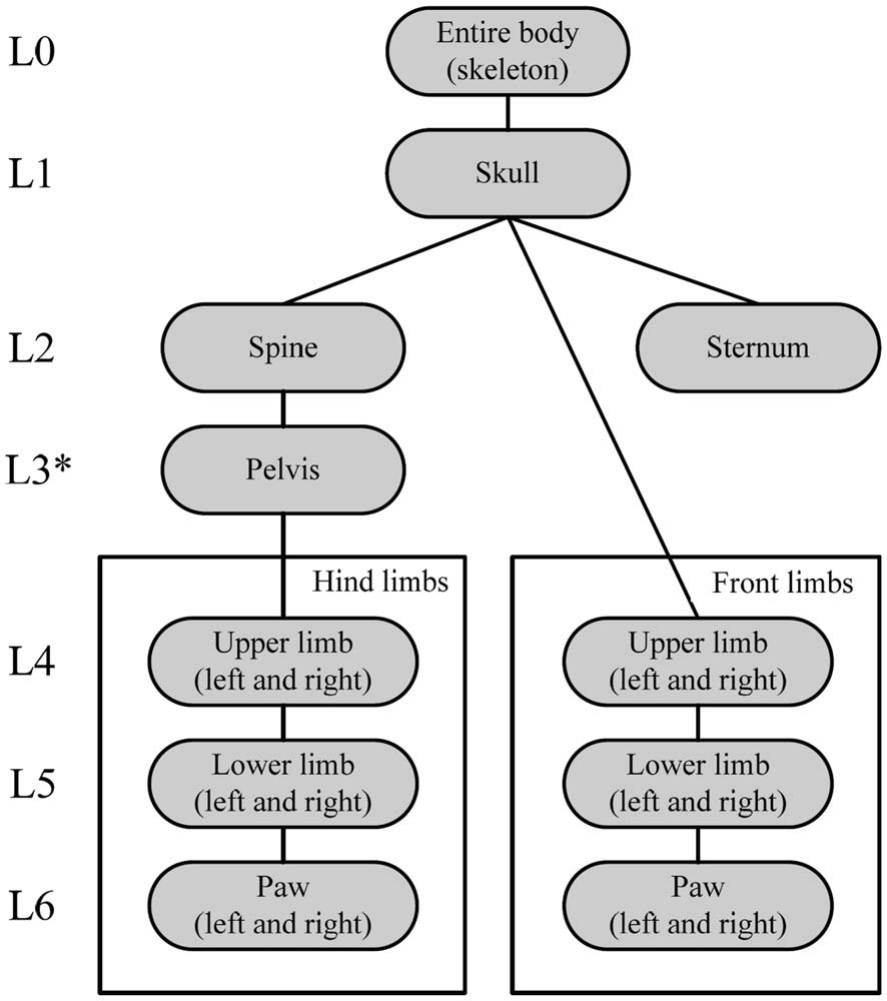
Hierarchical anatomical tree followed during the registration process. * indicates where user input is necessary: to pin-point the spine location where the vertebra connects the spine to the pelvis.

### Whole-body SPECT/CT Mouse Data Acquisition

In this study, we aim to demonstrate the robustness of the atlas-based segmentation with respect to the different whole-body SPECT scan settings and high posture variability that exists between those scans. Therefore, six male, *BALB/c* mice were retrospectively collected from several different imaging studies to represent that variability. One half was intravenously injected with 50±6 MBq ^99m^Tc-MDP, and the other one with 50±6 MBq ^99m^Tc-HDP. All mice were scanned 4 hours later using the Bioscan NanoSPECT/CT^™^ device (Washington DC, USA), equipped with four gamma cameras and pinhole apertures. With the combined scanner, the SPECT and CT were acquired one after the other without movement of the animal, so both imaging modalities are registered by hardware calibration. SPECT images were reconstructed using the ordered subset expectation maximization (OSEM) and CT images using the filtered back projection (FBP) algorithms. ^99m^Tc-MDP and ^99m^Tc-HDP are gamma-emitting radionuclide substances, where the metastable technetium (^99m^Tc) is tagged onto a phosphonate compound (MDP, HDP) to generate ^99m^Tc-MDP and ^99m^Tc-HDP respectively, which selectively concentrate in the bone and are the primary imaging agents used to image changes in bone vascularity and osteoblastic activity [20]. Both tracers are used in translational research. For all mice, part of the tracer is cleared by the liver and as such, this organ is visible as well. Between these six datasets, the resolution of the scanner varies, ranging from SPECT voxel size of 0.60×0.60×0.60 mm^3^ to 0.20×0.20×0.20 mm^3^. The highest resolution CT dataset has a voxel size of 0.10×0.10×0.10 mm^3^ and the lowest 0.99×0.99×1.00 mm^3^ (see Table 1 for further detail). All procedures involving animals were approved by the Animal Experimental Committee (DEC) of the Erasmus MC and performed in agreement with The Netherlands Experiments on Animals Act (1977) and the European Convention for Protection of Vertebrate Animals Used for Experimental Purposes (Strasbourg, 18 March 1986).

**Figure 5:**
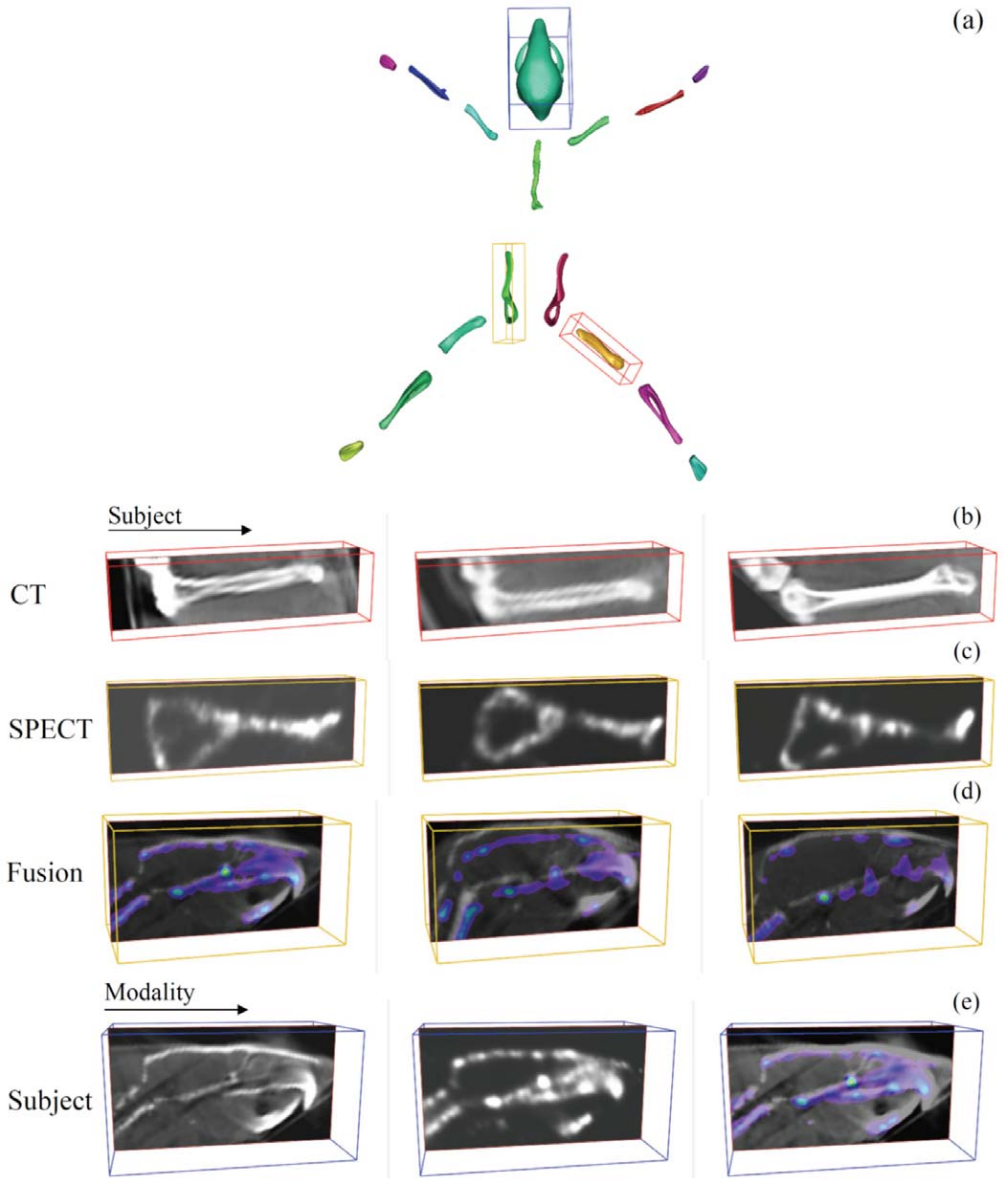
APR layout of the segmented mouse data. (a) - global articulated planar reformatted visualization of the atlas. (b), (c), (d) and (e) show the different data visualization options after applying the proposed approach. One can choose to visualize simultaneously and side-by-side a particular region of interest in cross-sectional studies for CT, SPECT or the combination of both. (b) - side-by-side visualization of the CT femur bone of 3 subjects, (c) - side-by-side visualization of the SPECT pelvic bone of 3 subjects, (d) - side-by-side visualization of the CT skull data fused with the correspondent SPECT data for 3 subjects, (e) - side-by-side visualization of the skull data of one particular subject: CT, SPECT and a combination of both. Follow-up data visualization was demonstrated in [10] for longitudinal CT mouse data.

The SPECT datasets were used for testing the proposed approach, and the correspondent µCT datasets to quantitatively validate the performance of the method.

**Table 2:** Mean Euclidian point to surface distance between the SPECT and CT skeletons after the pre-processing step.

	Mean Euclidian point to surface distance between the SPECT and correspondent CT skeletons and standard deviation (in voxels)
Mouse 1	6.4±11.1
Mouse 2	5.9±7.9
Mouse 3	4.5±5.7
Mouse 4	2.7±4.1
Mouse 5	9.6±13.4
Mouse 6	7.7±8.1
Mean	6.1±8.4

### SPECT/CT Data Pre-processing

To reduce the noise, small objects and other artifacts present in the SPECT data, in the first step of the algorithm, a threshold combined with a connected components filtering and morphological operations (erosion and dilation) was applied to the SPECT data to estimate the skeleton. Due to the variation of the tracer distribution, the extraction of the best possible skeleton requires minimal user input to adjust the threshold and morphological operators parameter settings (more specifically in the extraction of the spine centerline step, see section below). This results in a coarse estimation of the major accumulations of the radioactive tracer: bladder, kidneys, part of the liver and the skeleton. In Figure 3, one can see that due to the differences in nature between the SPECT and CT data, the resultant skeleton in the case of the SPECT data is incomplete, with several parts missing (especially the front limbs, hind limbs and the skull, which is incomplete with large holes). For the CT datasets on the other hand a simple threshold returns the full, complete skeleton (Figure 3).

**Table 3:** Quantitative results of the MOBY atlas-to-skeleton registration for 6 mouse SPECT datasets and [1].

	Mean Euclidian point to surface distance and standard deviation (in voxels)	
	Before registration	After registration
Mouse 1	10.3±10.1	2.4±2.4
Mouse 2	6.6±7.8	2.5±2.1
Mouse 3	8.2±8.2	2.0±1.7
Mouse 4	25.8±32.1	3.3±2.8
Mouse 5	10.4±8.7	2.6±1.9
Mouse 6	7.5±5.9	2.9±2.0
Mean	11.5±12.1	2.6±2.1
Baiker *et al.* [1] (µCT)	8.8±1.9	1.8±0.1

**Figure 6:**
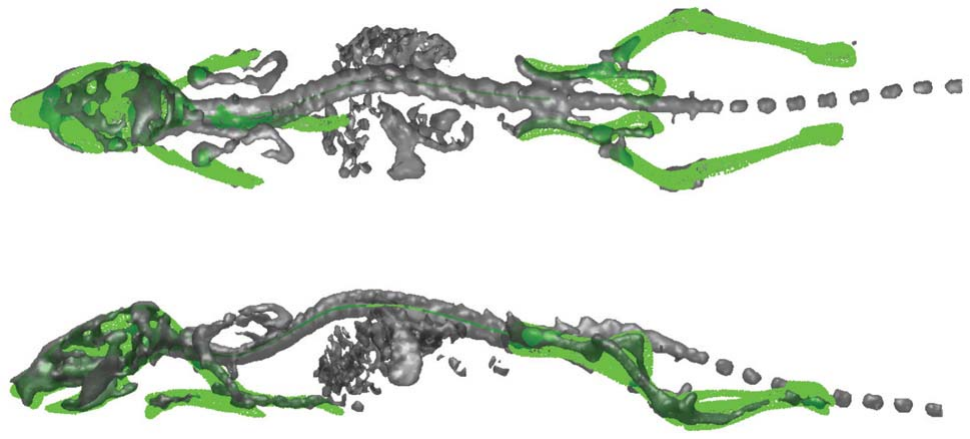
Top and side views of the segmented SPECT skeleton initially presented in Figure 1a). The registered MOBY atlas is represented in green.

### Articulated Atlas-based SPECT Skeleton Data Segmentation

In this step the articulated MOBY atlas is fitted to the skeleton extracted from the SPECT data. For this purpose a modified version of the fully automated approach presented in [1] was used. To deal with the large articulations between bones and/or bone groups, the registration of the atlas is employed following a hierarchical model tree (see Figure 4). It is initialized with a coarse alignment of the atlas and the entire target SPECT skeleton, where a similarity transformation model is applied to accommodate for the animal pose in the scanner and for size differences between animals (7 DoFs are taken into account: three for translation, three for rotation and one for isotropic scaling). After the coarse alignment of the entire skeleton, the individual bones are registered stepwise using the ICP algorithm [14], which is a method for point-based registration (skeleton surfaces in this case). We start at the skull, then, if necessary, the user locates the spine location where the vertebra connects the spine to the pelvis, the spinal centerline is extracted using three dimensional region growing until the pelvis is reached and registered and finally proceed to the back limbs, sternum and front limbs separately. The transformation models for the individual bones are dependent on the joint type (ball or hinge) and for each type a realistic motion model was defined ensuring that the bones remain in anatomically realistic shapes. See [1] and Movie S1 for more details.

### APR of Combined SPECT/CT Mouse Data

The fitted atlas yields a completely segmented SPECT skeleton where each bone has its own unique label. In this step we use the Articulated Planar Reformation (APR) algorithm [12, 13, Movies S2, S3, S4], that uses the segmented bones and the correspondent transformation models to reformat the data into segments corresponding to the atlas and thus maps the data to a standardized atlas space.

For each bone in the atlas, based on the surface representation of the bone and the corresponding linear transform that was determined during registration a bounding box is automatically determined. Using the bounding box, the volume SPECT data is resampled for each bone with the aim of obtaining the volume in a standard coordinate frame, which facilitates comparison (Figures 1(f, g), Movies S2, S3, S4).

The result is a visualization that consists of a global whole-body view at the top, with a number of focus views of longitudinal, cross-sectional or multimodal data side-by-side at the bottom (Figure 1(h) and Figure 5, Movies S3, S4). This standardized layout facilitates the comparison between subjects, eliminating large differences in animal posture. It allows the user to quickly identify regions/volumes of interest in the global whole-body view and then study the differences or changes in synchronized local per-segment focus views.

### Quantitative Evaluation Indices

To quantitatively validate the registration accuracy and enable comparison with the registration error achieved in µCT data as reported in [1], we used the same error metric to evaluate the SPECT segmentation: the mean Euclidean point to surface distance in voxels, *i.e.,* the shortest distance between objects in space. For each SPECT dataset, we calculated this distance between the registered MOBY atlas skeleton and the correspondent co-registered CT skeleton, before (*i.e.:* after the coarse alignment) and after articulated registration. The results were compared to the mean Euclidean point to surface distance published in [1] for µCT.

To investigate quantitatively intrinsic differences between SPECT and CT (Figure 3), we calculated the mean Euclidean point to surface distance between the estimated SPECT and its correspondent CT skeletons (Table 2).

## Results

Table 3 presents the Euclidian point to surface distance before and after articulated registration for all the subjects. For all the mice, after the registration the Euclidean point to surface distance between the MOBY atlas and the SPECT skeleton decreased: the calculated mean of the distance decreased from 11.5±12.1 to 2.6±2.1 voxels. In case of mouse 4 (Figure 3, bottom mouse), the Euclidean point to surface distance before registration is higher than for the other subjects for two reasons. The first reason is the positioning of the mouse in the scanner: the hind and front limbs were pulled towards the belly resulting in a lower alignment/overlay between the atlas and the data surfaces during the coarse alignment. The second reason is the fact that the SPECT skeleton after the pre-processing step in case of mouse 4 is underestimated (only remains of the limbs are visible, and a very small portion of the skull and the spine are present). Since the amount of total bone content of the skeleton has an influence on the coarse alignment step [1], in this case, during the coarse alignment, the atlas successfully accommodates for the animal position (prone/supine) and orientation of the animal, but the main overlap between the atlas and the animal after this coarse step happens between the skulls. Figure 3 shows three data examples with variations in posture with which the proposed method successfully coped. An example of a segmented SPECT dataset is presented in Figure 6 and Movie S1.

After applying the APR algorithm to both µCT and SPECT data, one can use a range of visualization techniques that enable exploration of both datasets, the result of which is depicted in Figure 5 and Movies S3, S4. The articulated layout visualization is shown, where all segments of the atlas have been spread out into a plane - Figure 5(a), Movies S3, S4. In Figure 5 and Movies S3, S4 the different visualization options are generated using the proposed approach. The femur, pelvis and skull were selected and are shown in the correspondent focus views. All focus views show an outline and an image slice visualization and one can visualize CT, SPECT, or a combination of both (where the SPECT data is shown as an overlay with a color map applied to it) in either cross-sectional analysis - Figures 5(b, c, d), Movie S3 or for multi-modality complementarity - Figure 5(e), Movie S4.

The entire articulated registration process was implemented in MATLAB R2008b^™^ and took approximately 2 minutes of runtime (including minimal user interventions to assist the spine centerline extraction) on a standard desktop PC (2.40 GHz Intel Quad Core^™^ with 3 GB of RAM, Windows^™^).

## Discussion and Conclusion

*In vivo* visualization of functional and anatomical information produces heterogeneous, high throughput data. Efficiently combining, analyzing and quantifying whole-body small animal cross-sectional, longitudinal and multi-modal data is a complex problem. In this paper, we demonstrated the feasibility of the articulated atlas-based skeleton segmentation approach combined with the articulated planar reformation algorithm for whole-body mouse bone imaging using SPECT.

Quantitative evaluation was performed by calculating the Euclidian point to surface distance between the registered atlas and the correspondent CT dataset. The obtained mean distance of 2.6±2.2 voxels, showed that the registration accuracy for the SPECT data is of the same order as the previously published results for µCT, 1.8±0.1 voxels [1]. The large difference in the standard deviation between the µCT fittings results and the ones presented in this paper might be due to the variable nature of the SPECT data (tracer uptake and distribution, where the tracer targets the bone growth and not the entire bone and partial volume effect) versus the more robust bone contrast in CT. Due to these factors the pre-processing step (extraction of the skeleton out of the data while removing the noise, small objects and other artifacts) may result in either a partial or a much thinner (Figure 3) or thicker skeleton than as seen in the µCT. This explains the difference in distance measures between the SPECT and CT skeleton surfaces (Table 2). As mentioned above, by collecting data from several different imaging studies, one of the goals of this study was to demonstrate the robustness of the atlas-based segmentation with respect to the different whole-body SPECT scan settings. Depending on the research question, the amount of injected tracer, the pinhole size and scan time a trade-off has to be chosen between resolution and signal. However, as long as a skeleton estimation is possible the approach presented here holds.

In [1], it was demonstrated that the proposed atlas-based segmentation method is robust with respect to osteolytic bone defects. Here, it was demonstrated, that the use of the articulated mouse atlas, with defined DoFs and size restrictions for each bone, proved to be robust for “incomplete” data (*i.e.:* images where large bits of limbs are missing), like exemplified in Figures 3 (bottom mouse) and 6. If a lower or an upper part of a limb is completely missing, than the proposed approach will only segment the part that is present in the dataset, *i.e.,* the part where there was significant/enough tracer uptake.

It also proved to be relatively insensitive to non-relevant objects still present in the image after threshold-based segmentation, like kidneys, bladder, some lung and liver. The proposed approach effectively compensated for the large variations in posture that existed within the data and yielded segmentation results requiring minimal user input. These were of satisfactory quality for the ensuing mapping of the data to the standard reference and side-by-side visualization. Applying the APR algorithm to multi-modal cross-sectional data proved to be useful to provide proper referencing and visualization for an intuitive exploration and comparison of µCT, SPECT data (Figure 5, Movies S3, S4). The authors are currently working on further extending the approach presented here to combine automatic segmentation of the different bones with tracer quantification.

The segmentation approach presented here was developed to cope with a scenario when a combined whole-body SPECT/CT bone scan is not always desired or available. Thus, one of the limitations of the proposed approach is the fact that the skeleton should exhibit sufficient image contrast, *i.e.,* direct application of the atlas fitting to SPECT data requires tracer uptake in the skeleton. When that is not the case, the limitation can be overcome by applying the fitting directly to the provided whole-body anatomical CT scan and then propagating it to the SPECT data. Furthermore, though very minimal, this method requires user input during the extraction of the possible skeleton out of the data. This only stands true when the method is applied to SPECT directly and correspondent CT data is not available. When CT whole-body data is available, due to its robust and consistent nature, this kind of user input is not required anymore, as shown and extensively validated in [1].

An articulated atlas-base skeleton segmentation method for SPECT whole-body small animal data was presented. The evaluation of the method demonstrated it to be sufficiently accurate and robust for intuitive exploration of whole-body, cross-sectional multi-modal small animal imaging data. The approach presented here can be applied to other animals, provided there is an adequate atlas.

## Supporting Information

Movie S1 **Atlas-based SPECT skeleton segmentation.** Demonstration of the articulated atlas-based small animal SPECT skeleton segmentation algorithm as described in the section **Articulated atlas-based SPECT skeleton data segmentation.**
(WMV)

Movie S2 **APR algorithm applied to mouse data.** Demonstration of the APR algorithm as described in the section **APR of combined SPECT/CT mouse data.**
(WMV)

Movie S3 **Side-by-side SPECT data exploration of 3 mice.** Exploration and visualization of cross-sectional SPECT mouse data of three different subjects after applying the APR algorithm as described in the **APR of combined SPECT/CT mouse data** and **Results** sections.
(WMV)

Movie S4 **Side-by-side SPECT/CT mouse data exploration.** Exploration and visualization of multi-modal, complementary SPECT/CT and the fusion of both mouse data after applying the APR algorithm as described in the **APR of combined SPECT/CT mouse data** and **Results** sections.
(WMV)
